# Ethical considerations in gene selection for reproductive carrier screening

**DOI:** 10.1007/s00439-021-02341-9

**Published:** 2021-08-23

**Authors:** Lisa Dive, Alison Dalton Archibald, Ainsley J. Newson

**Affiliations:** 1 Faculty of Medicine and Health, Sydney School of Public Health, Sydney Health Ethics, The University of Sydney, Level 1, Edward Ford Building A27, Sydney, NSW 2006 Australia; 2 Victorian Clinical Genetics Services, Murdoch Children’s Research Institute, Melbourne, VIC Australia; 3 Department of Paediatrics, The University of Melbourne, Melbourne, VIC Australia

## Abstract

Genetic carrier screening for reproductive purposes has existed for half a century. It was originally offered to particular ethnic groups with a higher prevalence of certain severe recessive or X-linked genetic conditions, or (as carrier testing) to those with a family history of a particular genetic condition. Commercial providers are increasingly offering carrier screening on a user-pays basis. Some countries are also trialing or offering public reproductive genetic carrier screening with whole populations, rather than only to those known to have a higher chance of having a child with an inherited genetic condition. Such programs broaden the ethical and practical challenges that arise in clinical carrier testing. In this paper we consider three aspects of selecting genes for population reproductive genetic carrier screening panels that give rise to important ethical considerations: severity, variable penetrance and expressivity, and scalability; we also draw on three exemplar genes to illustrate the ethical issues raised: *CFTR*, *GALT* and *SERPINA1*. We argue that such issues are important to attend to at the point of gene selection for RGCS. These factors warrant a cautious approach to screening panel design, one that takes into account the likely value of the information generated by screening and the feasibility of implementation in large and diverse populations. Given the highly complex and uncertain nature of some genetic variants, careful consideration needs to be given to the balance between delivering potentially burdensome or harmful information, and providing valuable information to inform reproductive decisions.

## Introduction

Genetic carrier screening for reproductive purposes has existed for half a century and has typically taken two forms. First, population-based screening has been offered to particular ethnic groups with a higher prevalence of certain severe recessive or X-linked genetic conditions (Hoedemaekers and Have 1998; Ioannou et al. 2010; Raz 2009). Second, carrier testing for those with a family history of a specific condition has been offered through clinical genetics services in many parts of the world, with new tests becoming available as relevant genes were identified. Such testing enabled those undergoing screening to determine their likelihood of having an affected child.

In recent years, as more genes associated with serious genetic conditions have been discovered and genetic testing technologies have advanced, the prospect of offering reproductive genetic carrier screening (RGCS) more broadly, rather than only to those known to have a higher chance^1^ of transmitting a rare genetic condition, has arisen (Antonarakis 2019; Delatycki et al. 2020; Rowe and Wright 2019). Fertility clinics may also offer RGCS to couples undertaking assisted reproduction, regardless of their family history (de Wert et al. 2021). Such initiatives have been underscored by increasing awareness of the benefits of offering carrier screening to anyone intending to have children (Henneman et al. 2016; McClaren et al. 2011). They are also supported by professional colleges and societies as well as patient support organizations (Edwards et al. 2015; Grody et al. 2013; Ong et al. 2018; RANZCOG 2019). A major rationale for population RGCS is that most children with severe recessive genetic conditions are born into families where there is no known family history.

While RGCS has been endorsed by medical colleges and professional societies, few jurisdictions to date have established formal publicly funded screening programs. In this absence, a burgeoning number of commercial providers have emerged. A comprehensive survey of commercial carrier screening is beyond the scope of this paper, but options include large companies offering screening globally, local carrier testing delivered through a clinical genetics service, or as part of assisted reproductive services.

The main current forms of carrier identification—ethnicity-based (or ancestry-based), clinical carrier testing, and commercial carrier testing products—demonstrate the increasing availability of information about genetic carrier status to inform reproductive decisions. However, these modes also generate inequities in access (Robson et al. 2020). Inequity is due both to the financial cost of the screening test for the person having it, as well as to variable awareness amongst healthcare providers (and consumers) regarding the potential benefits of RGCS (Archibald et al. 2017; Valente et al. 2020). This inequity provides strong grounds for advocating for better public funding of RGCS. Several governments have thus now begun to consider or implement an offer of RGCS as a publicly funded screening initiative (Schuurmans et al. 2019; Singer and Sagi‐Dain 2020).

While RGCS is ethically defensible, as it becomes more widely available a range of clinical, laboratory, economic, implementation and ethical considerations arise. These include determining acceptable program goals, how to implement screening equitably, and how to reflect community values (Dive and Newson 2021). Screening programs can raise different ethical and practical considerations compared to a clinical test offer. Population screening, by nature, is delivered at scale, with all program participants receiving the same intervention and little capacity to tailor to individual family histories. For RCGS, this means that considerations such as family and reproductive history—which would inform the approach to clinical testing—cannot always be factored in. Workforce and other resource considerations are important for a screening program’s feasibility and sustainability.

While the original criteria for population screening are only partially relevant to genetic screening programs (Andermann et al. 2008; Wilson and Jungner 1968), an adapted framework recommends a transparent approach to considering various trade-offs that must be made (Andermann et al. 2010). Genetic screening programs must uphold some of the fundamental criteria relevant to all population screening; for example, there should be evidence that the intervention is effective in responding to a defined need in the population, and the overall benefits of screening should outweigh any potential harms (Andermann et al. 2008). The clinical utility of results and the service delivery context, including the way participants are informed about the screening test, are also essential to consider (Molster et al. 2017; Pitini et al. 2019). Further, there are often dimensions of uncertainty to genetic test results (Newson et al. 2016). While uncertainty is not necessarily inherently harmful, it is important to consider how uncertainty may impact the utility of information gleaned from RGCS.

A fundamental challenge, which brings in all of these considerations, is how to approach the selection of genes to screen in population RGCS. Indeed, recommendations about deciding what to screen may not have evolved as quickly as testing technology, which has made it possible to screen for variants in many hundreds of genes at once (Wienke et al. 2014).^2^ The use of massively parallel sequencing in RGCS, especially among commercial providers, has led to increasingly large gene panels and higher rates of carrier detection. Some programs are returning results on a couples’ basis^3^ to safeguard program sustainability and ensure relevance of results.

In this paper, we consider three exemplar genes (*CFTR*, *GALT* and *SERPINA1*) and discern three factors (severity, variable penetrance and expressivity, and scalability) that together illustrate several ethical issues in selecting genes for large-scale RGCS initiatives. While we are not proposing that all variants within these genes be removed from RGCS, we argue that it is essential to consider how valuable the information provided through RGCS will be for those who will need to act upon it. We also argue that the issues raised warrant a cautious approach when deciding whether particular variants are included or excluded. This should be driven by screening principles that are informed by clinical considerations and cognisant of technological imperatives.

### Three genes that exemplify challenges in gene selection for reproductive genetic carrier screening

#### Example 1: CFTR

Alterations in the cystic fibrosis transmembrane conductance regulator (*CFTR*) gene can cause the autosomal recessive condition cystic fibrosis (CF). While most pathogenic variants in the *CFTR* gene are associated with so-called classic CF (chronic suppurative lung disease, pancreatic exocrine insufficiency and infertility), clinical presentations can vary significantly and can include mild phenotypes. Some of this variation is attributable to genotype.

Whilst milder *CFTR* variants are reported as ‘pathogenic’ because they can be disease causing, the extent to which an individual is clinically affected, if at all, can depend on the variant combination. So while *CFTR* is included almost universally on carrier screening panels, there can be practical and ethical complexities in choosing variants to report. A particularly challenging set of *CFTR* variants are the polyT alleles, including the 5T variant. For certain variant combinations, knowledge of the variant does not necessarily predict the chance of the person developing symptoms, nor their severity. This challenge is exemplified by the case of Sally and Pia (Box 1).

Box 1: CFTR Sally and Pia have one child, Ava (4 years) who was conceived via IVF. Their good friend Mark was the sperm donor. They have two embryos stored from their previous cycle. These embryos were conceived using Sally’s eggs and Pia carried the pregnancy. Sally (36 years) and Pia (40 years) would like to have another child and hope to use the stored embryos. They return to see their fertility specialist and are offered RGCS (which had not been offered to them previously). Sally and Pia are keen to know if there is a chance they could have a child with a serious genetic condition and after discussions with Mark, decide to go ahead.

Sally is surprised to learn that she is a carrier for the most common *CFTR* variant (p.F508del). Mark’s result shows that he is also a carrier, but for the 5T variant that can be more variable. Their fertility specialist refers them for genetic counseling. At this appointment, they learn that a child with the combination of variants that they carry could be unaffected, have CBAVD (congenital bilateral absence of the vas deferens) if male, or have mild CF symptoms.

Sally and Pia feel concerned by this information. They ask if their two stored embryos can be tested for these variants and are informed that unfortunately this is not technically possible. Starting a new round of IVF and having preimplantation genetic testing would come at significant financial cost. They are reluctant to use another sperm donor, as they are happy with Mark’s level of parenting involvement and do not want to introduce another person into the arrangement, or use Pia’s eggs as her age may impact their chances of viable pregnancy. They feel uncomfortable about the notion of prenatal diagnosis and possible termination of pregnancy when it is not certain that their future child’s health would be significantly impacted. Having been provided with this information, they feel strange doing nothing with it.

In the case example, a 5T variant is deemed to be pathogenic because it *can* be disease causing, but penetrance is incomplete (and low). In those who are affected, phenotypes are almost always mild and are determined in part by other variation on the same allele. There is substantial variation in clinical presentation associated with this allele and the accompanying uncertainty makes its inclusion in RGCS problematic. However, it is commonly reported through commercial panels, creating complex genetic counseling scenarios and difficult decision making for carrier couples.

There is broad consensus that the classic form of CF warrants the inclusion of the *CFTR* gene in reproductive carrier screening. However, it is less clear how milder *CFTR* variants should be managed with respect to carrier screening (Deignan et al. 2020), and it has been possible to avoid these through panel design. Yet as *CFTR* gene sequencing is now possible, the question of which variants to detect in the carrier screening context arises. If the standard clinical approach to report any disease-causing variants is used in when providing RGCS, it effectively transfers the challenge of interpreting the clinical significance of a variant combination to the clinician and to the person being screened. If used at population scale, this approach will have significant resource implications. Additionally, as Sally and Pia’s case shows, there is the potential for psychosocial impacts on couples. Alternative strategies in variant reporting may be needed, which can limit reporting to variant combinations that are associated with severe clinical presentations. Such an approach is being trialed in the Mackenzie’s Mission research study.

The *CFTR* gene illustrates the challenge of deciding whether to report variants that have incomplete penetrance and variable expressivity in the context of screening to inform reproductive decision making. *CFTR* is a gene that should be included in RGCS, but it also highlights the ethical complexity in deciding which variants to report when there are several dimensions of uncertainty. The likely value of information about some *CFTR* variants and their capacity to inform reproductive decision making should be assessed to determine whether they warrant inclusion in a screening panel. For those genes that have a wide range of variability in how they are expressed, it remains an open question as to whether such information is always beneficial (Silver and Norton 2021).

#### Example 2: GALT

Galactosemia is an autosomal recessive metabolic condition resulting in impaired ability to process the sugar galactose due to an enzyme deficiency. It presents in the newborn period with failure to thrive, susceptibility to infection, and liver dysfunction and can be fatal if not identified and treated. With early diagnosis and immediate intervention, severe impacts can be avoided. However developmental disability, movement disorders and speech problems may still occur. Additionally, females with classic galactosemia develop premature ovarian insufficiency.

Galactosemia is caused by alterations in the *GALT* gene. Most pathogenic *GALT* variants cause classic galactosemia as described above. However, a common *GALT* variant called the Duarte variant is associated with an attenuated phenotype. When an individual inherits a severe *GALT* variant (usually associated with classic galactosemia) and the Duarte variant, the clinical presentation is typically asymptomatic and is referred to as Duarte galactosemia (Fridovich-Keil et al. 2014). There is, however, some uncertainty over whether there are any other long term health impacts in adults. Individuals homozygous for the Duarte variant have 50% enzyme function and are asymptomatic. Nevertheless, the *GALT* gene is included on many commercial RGCS panels, with the Duarte variant reported as ‘pathogenic’.

Reporting variants that are not clinically significant, or which generate uncertainty for people screened, is problematic in the context of population RGCS. As screening becomes more available it will be offered more frequently by healthcare providers without specific genetics training—as Penny and Brad’s case (Box 2) illustrates. Whilst most healthcare providers have a degree of genomic competency, there are ongoing concerns about their capacity to communicate complex genomic information (Haga 2019; Hauser et al. 2018; Selkirk et al. 2013). Thus, cases such as the one outlined below—in which the result should not affect reproductive choices—can arise. Couple-based reproductive carrier screening, where the laboratory reviews the results of both reproductive partners and issues a combined couple report, can streamline the carrier screening process and reduce the possibility that results will be misinterpreted.

Box 2: GALT Penny is 34 years old and 12 weeks pregnant. She and her partner Brad were offered reproductive genetic carrier screening at their initial appointment with their obstetrician. Their obstetrician receives their results and informs them that both of their reports show that they are ‘carriers’ of variants in the *GALT* gene. She explains that there is a 1 in 4 chance this pregnancy will be affected by galactosemia and offers them prenatal diagnosis. They consent to CVS and the procedure is booked. The couple state that they would consider termination of pregnancy if the fetus is affected. They also ask how a baby with galactosemia might be impacted and their obstetrician offers to refer them for genetic counseling.

The genetic counselor reviews their results prior to the appointment and notes that both Penny and Brad’s individual carrier screening results show they carry the GALT Duarte variant. At the appointment the genetic counselor explains to Penny and Brad that although their carrier screening reports list them as ‘carriers’ for galactosemia, they both carry a common mild variant and people who inherit two copies of that variant do not have or develop galactosemia. Penny and Brad explain that they feel confused by the conflicting messaging but grateful to know that they have a low chance of having a baby with classic galactosemia. They cancel their CVS appointment.

#### Example 3: SERPINA1

The gene *SERPINA1* codes for the protein alpha-1 antitrypsin (AAT). AAT is produced in the liver and transported to the lungs where its role is to prevent damage to the lungs from infection and inhaled irritants (such as cigarette smoke). *SERPINA1* variants can lead to reduced AAT functionality, leading to alpha-1 antitrypsin deficiency, an autosomal recessive condition that can cause lung damage in adults and liver damage at any age. This can in turn lead to chronic obstructive pulmonary disease and/or bronchiectasis. Liver dysfunction can also occur due to accumulation of the abnormal AAT protein in the liver.

Alpha-1 antitrypsin deficiency is a highly variable condition, and this variability is due to environmental exposure and specific genetic variants in the individual. There are a range of *SERPINA1* alleles, the most common being ‘M’, ‘Z’, and ‘S’. The most common functional version of the protein is known as the M allele. The S allele is considered pathogenic because it causes reduced function and decreased serum AAT levels and can cause mild disease in adults when in combination with another pathogenic *SERPINA1* variant. The Z allele is the most common pathogenic *SERPINA1* variant and disrupts the function of the gene. The SZ genotype is associated with a slightly increased risk of COPD but not liver dysfunction.

Most people with alpha-1 antitrypsin deficiency are homozygous for the Z allele. In people with alpha-1 antitrypsin deficiency, lung disease generally starts in adulthood and is exacerbated by smoking and exposure to other lung irritants. Approximately 80–100% of people with alpha-1 antitrypsin deficiency will develop chronic obstructive pulmonary disease (Stoller et al. 2006). Whilst liver disease in people with alpha-1 antitrypsin deficiency can occur at any age, the most severe form occurs in infancy, resulting in severe liver damage and death in approximately 2% of children with the ZZ genotype.

As Ruby and Lee’s case (Box 3) illustrates, alpha 1 antitrypsin deficiency provides a good example of the ethical complexity in the notion of severity. The clinical presentation of this condition is both variable and unpredictable. It is however a gene with a relatively high carrier rate (Wienke et al. 2014). A phenotype of severe liver damage and death at a young age would be a reasonable inclusion for RGCS, yet this occurs infrequently and cannot be predicted with certainty. The majority of cases of alpha-1 antitrypsin deficiency are adult-onset, and can be mitigated to an extent by avoiding exposure to environmental lung irritants.

Box 3: SERPINA1 Ruby and Lee have a 3 year-old daughter Lian and have recently learned that Ruby is pregnant with their second child. Their general practitioner offers them carrier screening and their results have come back showing they are both carriers for the Z allele in *SERPINA1*. Their doctor tells them this means they have a 1 in 4 chance of having a child with alpha-1 antitrypsin deficiency. They are surprised to receive this news as they have no family history of the condition. The general practitioner explains this is a relatively common genetic condition. They search the internet and discover that in most cases this condition causes lung disease in adulthood but there is a small chance of severe liver problems in a child. They are immediately worried about Lian and ask to test her. The doctor explains that as she is healthy there is a low chance of her having liver problems. They find this reassuring but are worried about whether she will develop lung problems later in life. They are not sure about whether they want to know this information. Should they sit with the uncertainty or test her? They recognize that, if Lian does have alpha-1 antitrypsin deficiency, they can help reduce her exposure to environmental lung irritants but they are also concerned that this information may worry them and lead to an ongoing anxiety and hypervigilance with respect to her health. They also contemplate what their results mean for their current pregnancy, they are worried by the small risk of severe liver problems in an infant but are not sure if they feel comfortable ending a pregnancy based on this relatively small chance. They discuss how they feel about a child having an increased risk of developing serious lung problems as an adult and are conflicted about whether this is something they should avoid.

### Factors generating ethical issues

As the above three conditions illustrate, the inclusion of particular genes on reproductive genetic carrier screening panels can give rise to ethical issues. Here, we elucidate three significant factors to consider when designing RGCS panels: severity, incomplete penetrance and variable expressivity, and scalability.

#### Severity

With the technical capacity to screen a large number of genes, a key consideration in RGCS becomes how to choose which conditions are included in a screening panel. A key theme in the design of large-scale genetic carrier screening programs is the notion of seriousness or severity. For example, among the criteria applied to gene selection in the Australian Reproductive Genetic Carrier Screening project was that a condition should only be included if a typical couple “would take steps to avoid the birth of a child with that condition” (Kirk et al. 2021, p. 3).^4^

Developing screening panels in RGCS therefore requires a judgment about the potential impact of that condition on the affected individual and their family (Botkin 1995). Population-wide carrier screening programs are not focused on providing information about mild genetic conditions. Generally, only conditions that are deemed severe or life-limiting warrant inclusion in such a program, particularly when it is publicly funded (Inthorn 2014; Thomas et al. 2020). While a full exploration of the concept of severity is beyond the scope of this paper, it is important to recognize the ethical and practical complexity it generates in relation to gene selection.

There have been attempts to quantify the severity of genetic conditions, but such approaches only partially respond to the ethical complexity of deciding whether a condition is severe enough to warrant inclusion in RGCS. Lazarin et al (2014) draw on the expertise of nearly 200 health care professionals to identify a method for categorizing the severity of genetic conditions. They use an algorithm that involves identifying the condition’s clinical characteristics (for example impaired mobility, shortened life span, sensory impairments, etc.) and classifying the severity of each. The resulting algorithm allows for a generalisable scale for severity. This approach has been applied to RGCS panel design criteria, several of which reference different aspects of severity (Arjunan et al. 2020). This process enables a clarification of severity criteria that brings more consistency and objectivity to how the requirement for severity is interpreted in the context of gene selection for RGCS panels. While such tools are undoubtedly valuable, the examples of *CFTR* and *SERPINA1* demonstrate the difficulty of classifying a condition where there can be variability in presentation.

The decision as to whether a condition is severe enough to warrant inclusion in a screening panel has a strongly subjective element to it. The perception of a condition’s severity can be influenced by many different factors, and could differ depending on whose perspective is considered: a prospective parent with no known family history, a person with lived experience of the condition under consideration, a carer, a medical specialist who sees patients who have the condition, and so on. A further relevant consideration is the ongoing debate regarding how health, illness and disability should be construed, and the societal norms and other factors that affect perceptions of differences in health and ability (Reynolds 2020).

These factors point to a central issue, namely the value of the information generated from screening. In other words, will the couple be better off by virtue of having that information? That might be the case for conditions that clearly cause suffering (for example spinal muscular atrophy type 1), and that information might enable a couple to take steps to avoid having an affected baby. However in some other cases, such as those in our examples (see Boxes 1, 2 and 3) it might not be so obvious whether the information will be beneficial.

#### Incomplete penetrance and variable expressivity

Some decisions on which genes to include in RGCS panels have greater complexity because there can be significant variability in clinical presentation of the condition. Interpreting variants can be complex, especially when they are rare, and there may also be other contributing factors such as epigenetic effects, environmental impacts and modifier genes. Most genetic conditions vary in their expression, with some individuals less severely affected than others. This can include non-penetrance—that is, an individual may have a potentially disease-associated genotype without having any phenotype as a result. Such variable expressivity gives rise to uncertainty and is one element that can make it difficult to determine the implications of carrier screening results.

As noted above, an important consideration when reporting screening results is whether, and in what way, the information provided will be useful for participants. One of the central ethical trade-offs when making decisions about reporting variants is whether it would be worse for program participants if screening does not detect those with a high chance of having a child with a particular condition, or whether it would be more harmful for people to receive information about a variant that is highly complex and uncertain. Such information could potentially result in people with an increased chance of having a baby with a particular combination of variants taking possibly unnecessary steps to avoid that genotype. When variants associated with variable presentation are reported in RGCS, it can leave people with difficult choices based on information that has several dimensions of uncertainty or ambiguity. Such information can be highly burdensome, rather than helpful, for a family. Decisions such as whether to choose the complex and costly path of IVF with preimplantation genetic testing or, if a pregnancy is already underway, prenatal diagnosis with the possibility of termination of pregnancy, can be particularly challenging if the choice is based on a genetic test result with uncertain implications.

#### Scalability: reporting results at population scale

When a clinical or commercially available carrier test is scaled up to a population screening offer, some ethical considerations can be exacerbated due to the need to streamline the test offering and other logistical considerations. Scalability issues are over-arching and encompass other factors such as variable expressivity, however there are additional considerations regarding the design and implementation of a large-scale screening program. Here we consider how decisions about reporting results might differ in a screening program, the couples-based approach to screening, and the implications of non-genetic health care professionals delivering results.

An important component of RGCS program design is how reporting decisions should be made. Standard practice among commercial RGCS providers dictates that variants classified as ‘pathogenic’ or ‘likely pathogenic’ are reported. Currently, commercial RGCS providers have tended to adapt reporting frameworks from the diagnostic context for use in screening programs. This means that while a variant may be interpreted as ‘pathogenic’ or ‘likely pathogenic,’ some can be associated with milder clinical presentations or only cause the condition in combination with certain other variants. For example, when screening at scale, couples with an increased chance of having a child with the milder SZ genotype for the *SERPINA1* gene will be identified relatively frequently, impacting on screening program clinical resourcing and affecting the utility of the information for reproductive decision making. While variants associated with variable clinical presentations can be addressed in post-test genetic counseling in a diagnostic or clinical context, it may be more challenging in population screening—not least because a variant may have been identified in the absence of family history information, or with no known family history.

Currently, most commercially available RGCS provides each person tested with their own results rather than combining the results for a person and their reproductive partner together to form a couple-based result. The implication of this is that, at least where large gene panels are used, the majority of people who have RGCS receive a carrier result for one or more genes. This leaves the interpretation of the significance of these results to the healthcare provider who requested the screening. Such rates of carrier identification will generate resource implications, including laboratory scientists’ time in determining whether to report a variant, and genetic counsellors’ time to support each person informed of carrier status. Because RGCS—like all screening—is subject to resource constraints, some of the larger scale carrier screening programs—such as Mackenzie’s Mission (Dive and Newson 2021), and the Groningen trial (Schuurmans et al. 2019)—are reporting only couple-based results. Further to these practical considerations, reporting couple-based results reflects the aim of RGCS, namely to provide couples with information relevant to their reproductive decision making (De Wert et al. 2012). The evidence to date suggests that reporting couple-based results is deemed acceptable by most participants (Plantinga et al. 2019).

While a couples-based approach will reduce the number of increased chance results, for any variant that is reported even in a couple-based program, the health care provider will need to understand the identified variant(s) and interpret what the results mean for the couple. In the context of carrier screening programs at scale, current workforce limitations mean that health care professionals without specialized genetics training will have greater involvement in interpreting results and supporting couples to understand them. Therefore, another consideration when delivering a population screening program at scale is that RGCS will increasingly be ordered by non-genetics health care providers. It has been recommended that RGCS should always be provided in conjunction with genetic counseling, and ideally by a practitioner with genetics training (Sparks 2019), which might not be feasible in a large-scale program. If the clinical significance of the combination of particular variants—such as in examples 1 (*CFTR*) and 2 (*GALT*)—is not fully understood by the health professional reporting results, this can lead to misinterpretation of the significance of the variant combination for the couple. Receiving such information has the potential to be harmful by causing confusion or distress, could lead to unnecessary and burdensome reproductive interventions, and might not be useful for a couple in their reproductive decision making.

### Responding to ethical aspects of selecting genes and reporting results

The complexities outlined above show that for large-scale RGCS programs, it is important to be cognizant both of the value of the resulting information for screening participants as well as the implications of and distinctions between offering information about carrier status in clinical and population health contexts. Such factors should be major considerations in selecting which genes to screen for and which variants (or variant combinations) to report. It cannot be assumed that carrier information reported in a clinical context is necessarily appropriate to report in a population screening program. Additionally, RGCS programs should be mindful that the subjectivity of severity, genes with variable expressivity, and other complexities of interpretation become magnified in the context of publicly available carrier screening, particularly for conditions with lower population prevalence. Therefore, careful consideration needs to be given to the impact of a result that might be ambiguous or uncertain, or which might be resource intensive to return to an individual or couple. The example of Ruby and Lee (Box 3) shows how the information is not necessarily helpful in their reproductive decision making, and also raises concerns for their current child.

As a population screening program, RGCS should aim to identify couples most likely to have children with severe conditions, while minimizing uncertain or ambiguous results that have the potential to be harmful and to consume significant clinical resources. Reporting information about genetic variants that have variable expressivity or are only disease causing in combination with other variants might not be feasible or appropriate in a screening program, especially with less opportunity to tailor carrier testing to individual preferences and family histories. There are ethical trade-offs between giving information that might be more harmful than beneficial on the one hand, (as in the case examples noted in Boxes 1 and 2), and missing a potentially valuable finding on the other.

Scientific understanding of genetic variants and their impact on health is constantly evolving. Therefore, revising gene lists is also an essential component of population RGCS. A condition not previously included might warrant inclusion on a screening panel if it becomes better understood. Similarly, it is possible that increased knowledge about a gene or condition could justify its removal from a RGCS program. As genes are reinterpreted and reclassified, the issue of whether or how to convey this information to previous program participants is complex and requires cautious consideration and planning (Silver and Norton 2021).

One option to alleviate some of the ethical concerns for RGCS might be to adopt smaller gene panels and to exclude variants where factors like the three we have discussed arise. This will necessitate shifting from a perspective that screening is designed or interpreted as an opportunity to ‘find everything’, to one where screening is designed to detect a well curated and reliable list of conditions that meet requirements including resource conservation in reporting results, ease of result delivery (including by non-specialist health professionals) and value of the information for couples. A conservative approach that is feasible to deliver at scale—across a population—and which only reports variants (or combinations of variants) that will be valuable for reproductive decision making or intervention very early in life^5^ should be the default option, with any departures from this clearly justified. This may mean that current variant reporting guidelines, which evolved out of the diagnostic setting, may need to be modified for the carrier screening context.

## Conclusion

RGCS is an important intervention that can enhance reproductive autonomy. However as RGCS becomes more widely available, increasingly as part of publicly funded programs, decisions about what genes to include on screening panels and how to interpret and report results can become more difficult. The requirement for large public screening programs to be delivered at scale means that current approaches used in the clinical context or by commercial testing providers might not be suitable. We have discussed variants in three exemplar genes (*CFTR*, *GALT* and *SERPINA1*) and discerned three factors (severity, variable penetrance and expressivity, and scalability) that together generate ethical complexity in gene selection. We have argued that these factors warrant a cautious approach to screening panel design; one that takes into account the likely value of the information generated by screening, and the feasibility of implementation in large and diverse populations. Regular review of screening panels is also imperative. Given the highly complex and uncertain nature of some genetic variants, careful consideration needs to be given to the balance between delivering potentially burdensome or harmful information, and providing valuable information to inform reproductive decisions.

## Data Availability

Not applicable.

## Footnotes

1 In this paper, we use the term ‘chance’ rather than ‘risk’ to denote the probability of a child being born with a particular genetic condition. We also use ‘increased chance’ rather than ‘high chance’. This terminology is gaining increasing traction in discussions of various reproductive and antenatal screening interventions in Australia, and is reflected in some professional guidelines. The impetus for this change is to recognize the potentially pejorative undertones of terms like ‘high’ and ‘risk’ rather than the more neutral ‘increased’ and ‘chance’, and the subtle but important message this may provide regarding people’s reproductive choices.

2 This is often referred to as expanded carrier screening or expanded universal carrier screening.

3 In this paper we will refer to the reproductive decisions undertaken by women, couples or families. In so doing, we take “women” to mean anyone who is able to become pregnant, whether or not they identify as a woman; we also take “couples”, “(intended) parents” and “families” to include a broad range of non-heterosexual couples and other family structures who might seek to have a child, including those who will use donor gametes and two reproductive partners, who may or may not be a married (or de facto) “couple”.

4 We acknowledge the empirical limitations of exercising this criterion, including that objective evidence would not be available for all conditions. How influential a criterion set at the level of individual couples should be in the design of a population screening initiative also requires further discussion.

5 In Mackenzie’s Mission, certain genes were included even where intervening to prevent a birth may be contentious, because an intervention very early in life can be beneficial but would need to occur before the results of newborn bloodspot screening are known (Kirk et al. 2021).
